# Supplementary material for: Inverted valley polarization in optically excited transition metal dichalcogenides
Source: Nat Commun. 2018 Mar 6;9:971. doi: 10.1038/s41467-018-03354-1 (PMC5840402; doi:10.1038/s41467-018-03354-1)
Supplement: Supplementary file 1 — Supplementary Information [file 41467_2018_3354_MOESM1_ESM.pdf]

Supplementary Information: Inverted valley polarization in optically excited transition metal dichalcogenides

Gunnar Berghäuser[1]* and Ivan Bernal-Villamil[1], Robert Schmidt[2], Robert Schneider[2], Iris Niehues[2], Paul Erhart[1], Steffen Michaelis de Vasconcellos[2], Rudolf Bratschitsch[2], Andreas Knorr[3], and Ermin Malic[1]
1 *Department of Physics, Chalmers University of Technology, Gothenburg, Sweden,*
2 *Institute of Physics and Center for Nanotechnology, University of Münster, Münster, Germany,* 3 *Institut für Theoretische Physik, Technische Universität Berlin, Berlin, Germany*

SUPPLEMENTARY NOTE 1: PUMP-PROBE EXPERIMENT

Creation of the pump and probe pulses

The pump-probe experiments are performed with a laser system based on a femtosecond-pulsed fiber laser with a wavelength of 1550 nm and a repetition rate of 40 MHz. Two pulse trains (pump and probe) of the laser are converted into near infrared supercontinua (NIR) using highly nonlinear fibers. The NIR supercontinua are converted into visible radiation by second harmonic generation (SHG) [1]. The pump pulses have a spectral bandwidth of 8 meV and are peaked at the denoted energy. For probing the B exciton, the probe pulse has a spectral bandwidth of 250 meV and is centered at 2.32 eV, while for probing the A exciton it is at 1.98 eV.

Simultaneous pump-probe measurement of the unpumped and pumped valley

The K valley of the monolayer is pumped by a left circularly polarized laser pulse, while the probe pulse has a linear polarization. This way, we can simultaneously measure the transient differential transmission of the K and K' valleys (Supplementary Fig. 1). The linear polarization, being a superposition of left and right circularly polarized light, probes both valleys simultaneously. A quarter wave plate (QWP) converts the two circular polarization components into two orthogonal linear polarization components with a $\lambda/4$ phase shift. The pump laser is blocked by a longpass (LP) or shortpass filter (SP), depending on the type of measurement. A calcite beam displacer (BD) placed behind the lens (L) vertically displaces the two orthogonal linear polarization components. Hence, they are imaged onto different lines of the CCD for parallel readout of the spectra with the multi-track mode of the CCD.

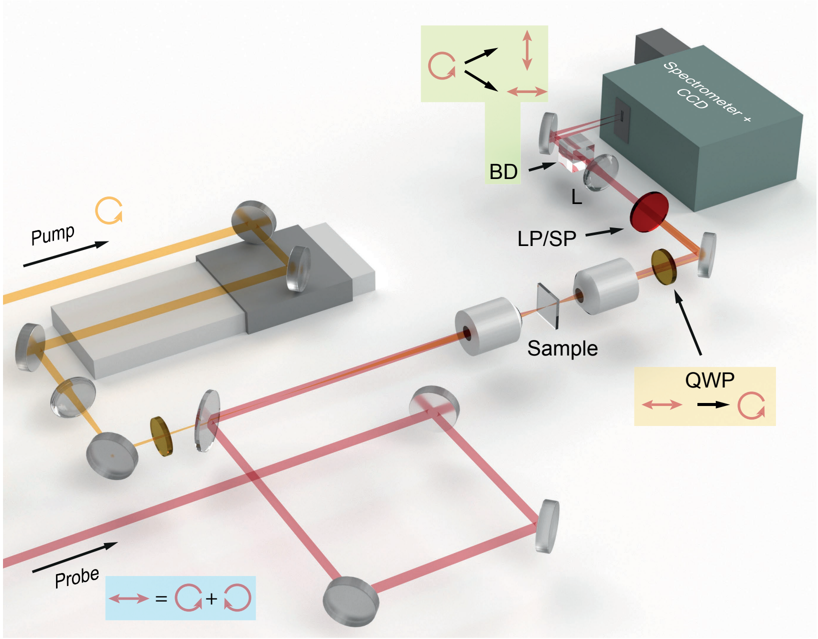

Supplementary Figure 1: Schematic drawing of the optical setup.

SUPPLEMENTARY NOTE 2: TRANSIENT TRANSMISSION SPECTROSCOPY ON MONOLAYER WS_2

Linear absorption spectrum of the investigated WS_2 monolayer

Supplementary Figure 2 shows the white light absorption spectrum of the WS_2-monolayer. The linewidths of the A and B exciton are extracted by fitting the absorption spectrum with a superposition of Gaussian functions.

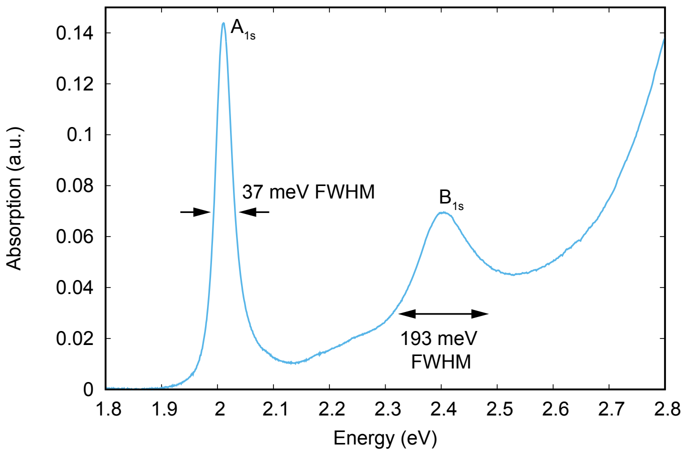

Supplementary Figure 2: Linear absorption spectrum of the investigated WS_2-monolayer.

Dynamics of the A excition

The left column of Supplementary Fig. 3 shows the population dynamics of the A exciton in the pumped K valley (denoted as A) and the unpumped K' valley (denoted as A') for different excitation energies. The population dynamics are extracted from the spectrally resolved pump-probe measurements by integrating the A exciton signatures along the spectral direction. This removes renormalization and spectral broadening effects from the measurements, yielding a signal which only depends on the bleaching of the exciton resonance and is proportional to the population of excitons at the K and K' points [2].

From the population dynamics, we calculate the valley polarization degree P_{v} (Supplementary Fig. 3, right column) as $P_{\mathrm{v}} = (N^K - N^{K'})/(N^K + N^{K'})$. Here, N^K and $N^{K'}$ are the A exciton populations in the K and K' valleys.

When the K valley is pumped near the A exciton resonance (Supplementary Fig. 3(a)), a strong population of A excitons is created by the pump laser in the K valley, while less excitons are created in the K' valley. This results in a valley polarization degree of 0.6 at zero delay time. For longer delay times, the K' valley is populated due to intervalley scattering processes leading to a decay of the valley polarization within a few picoseconds. Moving the energy of the pump laser towards the B exciton resonance (Supplementary Fig. 3(b-d)) increasingly equalizes the population of A excitons in the K and K' valleys due to the resonant Dexter-like intervalley coupling mechanism and results in a reduced valley polarization degree at zero delay time at these excitation energies. In contrast to the initial valley polarization, the decay time of the valley polarization degree is almost constant for different excitation energies.

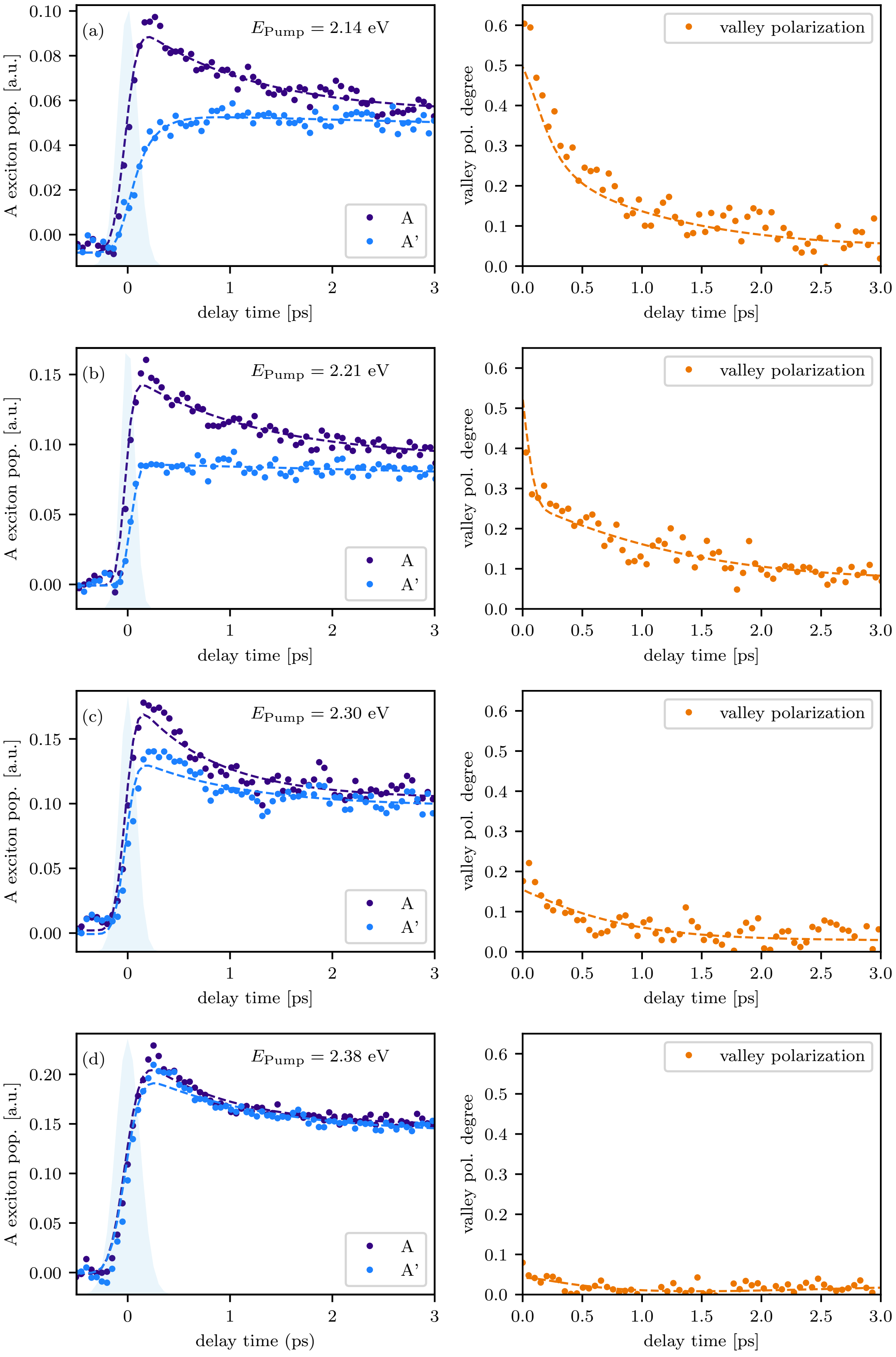

Supplementary Figure 3: **Measured population dynamics of the A exciton and A valley polarization.** Population dynamics of the A excitons in the pumped K and unpumped K’ valley (left column) and the corresponding transient valley polarization degree (right column) for different excitation energies. As the excitation energy moves closer to the B exciton resonance, the valley polarization degree at zero delay time decreases due to the Dexter-like intervalley coupling mechanism. The light blue shaded areas show the instrument response function of the ultrafast pump-probe setup. For all measurements a pump fluence of $6.5\mu\mathrm{J/cm}^2$ is used.

Dynamics of the B exciton

The B exciton population dynamics is measured for excitation of the K valley at or near the A exciton resonance with a pump fluence of $20\mu\mathrm{W/cm}^2$. Supplementary Figure 4 shows the B exciton population dynamics for both valleys denoted as B and B'. The populations and valley polarization degrees are extracted from the pump-probe measurements in the same manner as for the A excitons. The B exciton signature overlaps with the high energy wing of the stronger A exciton. To extract the correct B exciton populations, it is important to integrate only over the pump-probe signature belonging to the B exciton. Hence, the A exciton contribution is fit by a Voigt function and subtracted from the spectra prior to integration. Due to the limited spectral bandwidth of the measurement, this subtraction is prone to uncertainties and determines the width of the error bars of the extracted absolute valley polarization degrees.

Supplementary Figure 4(a) shows that pumping the K valley at the A exciton energy creates a strong population of B excitons in the K' valley at zero delay time while there is only a small population in the K valley. In this case, the Dexter-like intervalley coupling between A excitons in the K valley and B excitons in the K' valley creates a strong inverted valley polarization which decays within a few picoseconds (Supplementary Fig. 4(b)).

Pumping slightly off-resonant on the high-energy side of the A exciton (Supplementary Fig. 4(c) and (d)), reduces the efficiency of the Dexter-like intervalley coupling. Therefore, the initial valley polarization at zero delay time is reduced with respect to the resonant case.

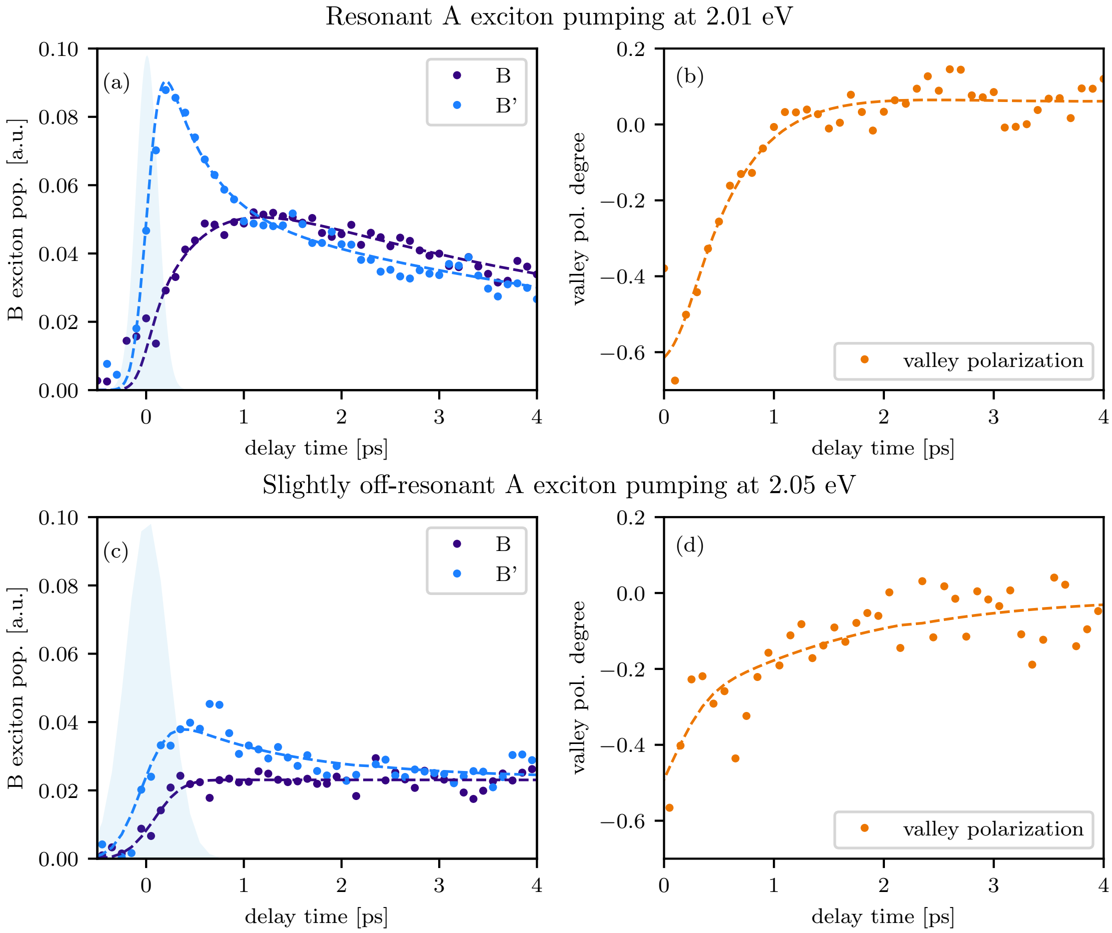

Supplementary Figure 4: **Measured population dynamics of the B exciton and B valley polarization** Population dynamics of the B excitons in the pumped (B) and unpumped valley (B') and transient valley polarization degree. The light blue shaded areas show the instruments response functions of the pump-probe setup.

SUPPLEMENTARY NOTE 3: COHERENT INTER STATE COUPLING

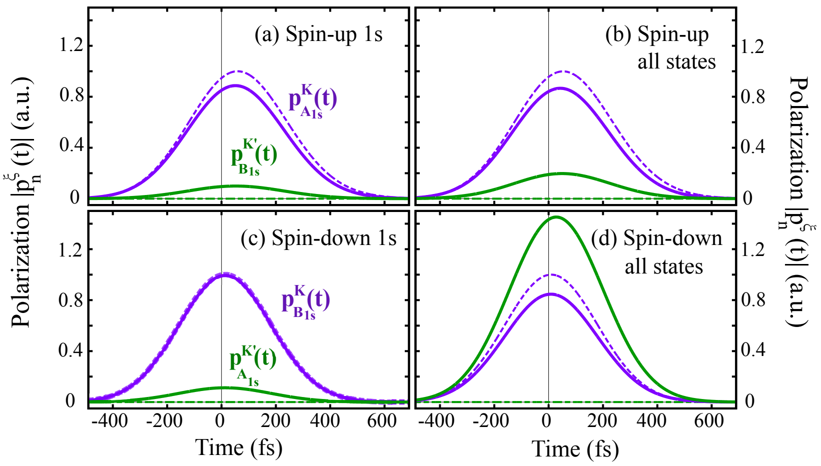

Supplementary Figure 5: **Calculated excitonic polarizations in pumped and unpumped valley.** Temporal evolution of the absolute value of the exciton polarization $p_n^{\xi_s}(t)$ for spin-up ((a) and (b)) and spin-down ((b) and (d)) states after resonant excitation of $\mathrm{A_{1s}}$ and $\mathrm{B_{1s}}$ exciton in the K valley, respectively. The optically driven states (purple lines) couple via Dexter interaction to the states of the same spin in the unpumped K' valley (light blue lines). The thin dashed lines show the case when the intervalley coupling is turned off. In (a) and (c), only 1s states are included in the calculation, which leads to a comparable microscopic polarization in the unpumped K' valley for both spin-systems. In (b) and (d), higher excitonic states are also included and the sum of all polarizations for each spin direction $\sum_n |p^{\xi(\bar{\xi})}_{A(B)_n}(t)|$ is shown. Here, we find a much stronger intervalley coupling of spin-down states. This is due to the nearly resonant Dexter coupling between the optically excited $\mathrm{B_{1s}}$ state and the higher excitonic $\mathrm{A_{ns}}$ states in the unpumped valley.

Supplementary Figure 5 illustrates the temporal evolution of the absolute value of the excitonic polarization $|p_n^{\xi_s}(t)|$ after excitation with a temporally broad (200 fs) and spectrally narrow (20 meV) pulse tuned at the frequency of either the $\mathrm{A_{1s}}$ or $\mathrm{B_{1s}}$ excitonic state in the K valley. For A excitons, we use the homogeneous dephasing of the microscopic polarization $\gamma_{hom} = 10\,\mathrm{meV}$ at room temperature [3]. The higher energetic position of the B exciton allows for more relaxation channels, which leads to a faster decay of the B states. This results in much broader resonances in linear absorption ($\gamma_{hom} = 50\,\mathrm{meV}$), cf. Supplementary Fig. 2. The dashed lines in the background correspond to the polarization without intervalley coupling. Here, we only see the optically induced polarizations in the K valley (purple lines). As soon as the Dexter coupling is switched on, we find a non-zero excitonic polarization for both spin systems also in the unpumped valley (light blue lines). At the same time, the amplitude of the optically induced polarizations is slightly reduced, where the effect is more pronounced for the spin-up system due to the narrower linewidths of A excitons. Taking into account also higher excitonic $\mathrm{A_{ns}}$ and $\mathrm{B_{ns}}$ states (Figs. 5(b) and (d)), the oscillation transfer to the unpumped K' valley becomes more pronounced for the spin-up system due to additional states for the coupling. However, overall the effect is rather small, demonstrating the predominant coupling between the energetically lowest $\mathrm{A_{1s}}$ and $\mathrm{B_{1s}}$ excitons, cf. Supplementary Fig. 5(b). In contrast, for the spin-down system, we observe a drastic increase in the amplitude of $|p_A^{K'}(t)|$, which clearly exceeds the optically driven polarization $|p_B^K(t)|$ in the K valley, cf. Supplementary Fig. 5(d). This can be traced back to the stronger optical oscillator strength of the $\mathrm{B_{1s}}$ excitons and a large number of higher excitonic $\mathrm{A_{ns}}$ states in the unpumped valley that are nearly resonant to the optically excited $\mathrm{B_{1s}}$ state. As a result, the Dexter coupling is much stronger than in the spin-up case.

SUPPLEMENTARY NOTE 4: ESTIMATION OF DEXTER LIKE TRANSFER FOR DIFFERENT TMDS

Here we show how to estimate the Coulomb induced intervalley transfer for different TMDs. As discussed in the main text one can for the intervalley coupling exploit the large distance between the valleys, e.g. $|\mathbf{k}-\mathbf{k}'+\mathbf{\Delta}_{\mathrm{KK}'}| \approx \mathbf{\Delta}_{\mathrm{KK}'}$. In this limit the coupling constant reduces to the two dimensional coulomb potential $V_q^{\mathrm{2D}} = e^2/(2\varepsilon_0 q)$ where e is the electron charge and ε_0 the vacuum permittivity and the excitonic wave functions $\sum_{\mathbf{k}} \theta_{\mathbf{k}}^{1s\xi_s} = \theta^{1s\xi_s}(r=0) = \sqrt{\frac{2}{\pi}\frac{1}{\mathrm{a_B}}} \approx 0.6\,\mathrm{nm}^{-1}$, where we have used an excitonic Bohr radius of 1 nm. Exploiting this long range limit one can estimate the intervalley coupling constants for different TMDs using the excitonic Bohr radius $\mathrm{a_B}$, the lattice constants a_0 and the substrate screening ϵ_s. Having estimated the coupling constant for different TMDs one can easily find the relative oscillation transfer ($p_{21} = 1/(1/v_{21}+1)$) determined by the ratio

between direct and indirectly excited polarizations $v_{21} = p_2/p_1$. For the case of resonant excitation of the p_1 this is given by the coupling constant, the spin orbit coupling of the material and the substrates dielectric constant. The later can be used as tuning node for the coupling. The results for four different TMDs (WS_2, WSe_2, MoS_2 and $MoSe_2$) are shown in Supplementary Fig. 6. The intervalley transfer is inversely proportional to the spin orbit coupling and directly proportional to the lattice constant. A large lattice constant and a small spin orbit coupling supports the intervalley coupling. Furthermore one can tune the coupling using different substrates characterized by different dielectric constants. For the shown TMDs the largest effect can be seen for MoS_2 characterized by a spin orbit coupling of SOC $= 151\,\mathrm{meV}$ and a lattice constant of $a_0 = 0.316\,\mathrm{nm}$. Next in line is $MoSe_2$ with SOC $= 215\,\mathrm{meV}$ and $a_0 = 0.33\,\mathrm{nm}$. The tungsten based TMDs show a smaller coupling constants, here the in the main text discussed WS_2 (with $a_0 = 0.316$ and SOC $= 396\,\mathrm{meV}$) shows a slightly larger transfer than WSe_2 (with $a_0 = 0.332$ and SOC $= 501\,\mathrm{meV}$).

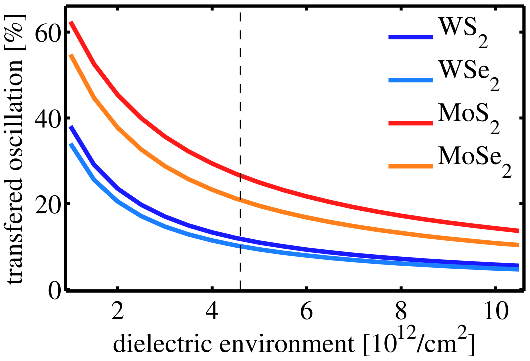

Supplementary Figure 6: **Estimated intervalley transfer for different TMD and substrate dielectrica** The relative intervalley transfer for resonant excitation is inversely proportional to the substrates dielectric constant.

---

* Electronic address: `gunbergh@chalmers.se`

SUPPLEMENTARY REFERENCES

[1] K. Moutzouris, F. Adler, F. Sotier, D. Träutlein, and A. Leitenstorfer, Multimilliwatt ultrashort pulses continuously tunable in the visible from a compact fiber source, Optics Letters **31**, 1148-1150 (2006), ISSN 0146-9592.

[2] R. Schmidt, G. Berghäuser, R. Schneider, M. Selig, P. Tonndorf, E. Mali, A. Knorr, S. Michaelis de Vasconcellos, and R. Bratschitsch, Ultrafast Coulomb-Induced Intervalley Coupling in Atomically Thin WS_2, Nano Letters **16**, 2945-2950 (2016).

[3] M. Selig, G. Berghäuser, A. Raja, P. Nagler, C. Schüller, T. F. Heinz, T. Korn, A. Chernikov, E. Malic, and A. Knorr, Excitonic linewidth and coherence lifetime in monolayer transition metal dichalcogenides, Nature Communications **7**, 13279 (2016).
